# Nanoscale control of competing interactions and geometrical frustration in a dipolar trident lattice

**DOI:** 10.1038/s41467-017-01238-4

**Published:** 2017-10-17

**Authors:** Alan Farhan, Charlotte F. Petersen, Scott Dhuey, Luca Anghinolfi, Qi Hang Qin, Michael Saccone, Sven Velten, Clemens Wuth, Sebastian Gliga, Paula Mellado, Mikko J. Alava, Andreas Scholl, Sebastiaan van Dijken

**Affiliations:** 1 Advanced Light Source, Lawrence Berkeley National Laboratory (LBNL), 1 Cyclotron Road, Berkeley, CA 94720 USA; 2 COMP Centre of Excellence, Department of Applied Physics, Aalto University, P.O. Box 11100, Espoo FI-00076 Aalto, Finland; 3 Molecular Foundry, Lawrence Berkeley National Laboratory (LBNL), 1 Cyclotron Road, Berkeley, CA 94720 USA; 4 Dipartimento di Fisica, Università di Genova, via Dodecaneso 33, I-16146 Genova, Italy; 5 NanoSpin, Department of Applied Physics, Aalto University School of Science, P.O. Box 15100, FI-00076 Aalto, Finland; 6 Department of Physics, University of California, Santa Cruz, CA 95064 USA; 7 Materials Sciences Division, Lawrence Berkeley National Laboratory, 1 Cyclotron Road, Berkeley, CA 94720 USA; 8 Institut für Nanostruktur- und Festkörperphysik, Universität Hamburg, Jungiusstrasse 11, 20355 Hamburg, Germany; 9 Center for X-ray Optics, Lawrence Berkeley National Laboratory, 1 Cyclotron Road, Berkeley, CA 94720 USA; 10 Daegu Gyeongbuk Institute of Science and Technology (DGIST), 50-1 Sang-ri, Hyeonpung-myeon, Dalseong-gun, Daegu, 42988 Republic of Korea; 11 SUPA, School of Physics and Astronomy, University of Glasgow, Glasgow, G12 8QQ UK; 12 School of Engineering and Sciences, Adolfo Ibáñez University, Diagonal Las Torres, 2640 Peñalolén, Santiago Chile

## Abstract

Geometrical frustration occurs when entities in a system, subject to given lattice constraints, are hindered to simultaneously minimize their local interactions. In magnetism, systems incorporating geometrical frustration are fascinating, as their behavior is not only hard to predict, but also leads to the emergence of exotic states of matter. Here, we provide a first look into an artificial frustrated system, the dipolar trident lattice, where the balance of competing interactions between nearest-neighbor magnetic moments can be directly controlled, thus allowing versatile tuning of geometrical frustration and manipulation of ground state configurations. Our findings not only provide the basis for future studies on the low-temperature physics of the dipolar trident lattice, but also demonstrate how this frustration-by-design concept can deliver magnetically frustrated metamaterials.

## Abstract (web summary)

Artificial magnetic nanostructures enable the study of competing frustrated interactions with more control over the system parameters than is possible in magnetic materials. Farhan et al. present a two-dimensional lattice geometry where the frustration can be controlled by tuning the unit cell parameters.

## Introduction

Artificial square ice^1^, consisting of dipolar coupled Ising-type nanomagnets, lithographically arranged onto a two-dimensional square lattice, was introduced as a two-dimensional analog to pyrochlore spin ice^2^ and provided the prospect to directly explore the consequence of geometrical frustration using appropriate imaging techniques^3–7^. However, due to imbalanced competing interactions of nanomagnets meeting at the four-nanomagnet vertices, the spin-ice degeneracy in artificial square ice is lifted^8^ and the system lacks the macroscopic degeneracy of its three-dimensional counterpart, the pyrochlore spin ice. Indeed, artificial square ice, when exhibiting thermally driven moment fluctuations, has been shown to have a clear pathway toward a long-range ordered ground state configuration^9–12^. Several concepts have been proposed to overcome this problem, most prominently by introducing a height offset between the corresponding nanomagnets^8, 13–15^. However, a realization that is at the same time technically simple and appropriate for direct real-time imaging of thermal fluctuations remains elusive^15^. Alternatively, highly frustrated artificial kagome spin ice has been extensively investigated, as it exhibits some analogy to pyrochlore spin ice^16^, including a spin liquid phase with short-range correlations^17–21^. Still, long-range dipolar interactions have been shown to overcome the fixed degree of frustration at low-temperature regimes, leading the system to access a long-range ordered ground state^17–19^.

This raises the question whether a two-dimensional geometrical concept can be proposed that shares some similarities to the square ice geometry, while exhibiting a higher degree of geometrical frustration.

In the following, we address this point by exploring moment configurations achieved in a two-dimensional artificial frustrated system consisting of nanomagnets occupying the sites of a so-called trident lattice. Following thermal annealing, we observe how accessible low-energy configurations can be directly manipulated by tuning the balance of competing interactions.

## Results

### The dipolar trident lattice

**Fig. 1:**
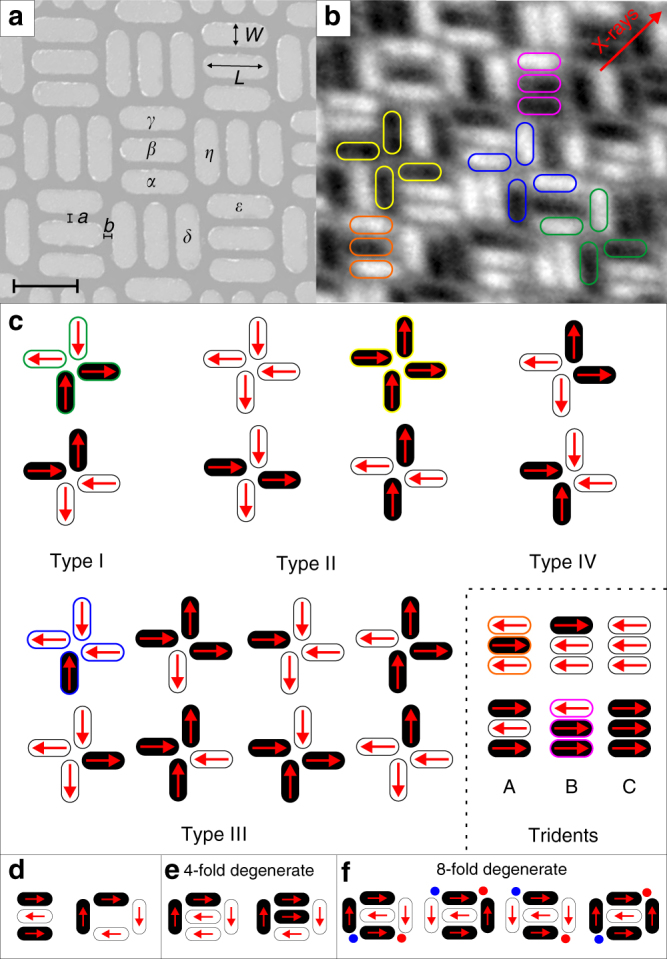
The dipolar trident lattice. **a** Scanning electron microscope image of a dipolar trident lattice (*a* = *b* = 50 nm, *L* = 450 nm, *W* = 150 nm). The black scale bar indicates a length of 450 nm. **b** X-ray magnetic circular dichroism (XMCD) image resolving moment configurations achieved in the trident lattice. Nanomagnets with a magnetization pointing toward the incoming X-ray propagation vector (indicated by a red arrow) appear dark, while moments opposing that direction appear bright. **c** Vertex and trident types listed with increasing energy. In both **b**, **c** green, yellow, and blue frames highlight the realizations of Type I, Type II, and Type III vertices, respectively. Regarding tridents, Type A and B tridents are highlighted with orange and magenta frames, respectively. **d** Minimization of dipolar interactions in an isolated trident building block would result in a Type A domination. On the other hand, nearest-neighbor nanomagnets at the four-nanomagnet vertices will prefer a head-to-tail moment alignment, which would result in clockwise or anti-clockwise vortices. **e** Satisfying vertex interactions (creation of vortices or Type I vertices) results in frustration of trident moments. **f** Satisfying trident interactions (creation of Type A tridents) results in two of nearest-neighbor vertex moments being aligned head-to-head (red circles) or tail-to-tail (blue circles)

We introduce an artificial frustrated system consisting of three-nanomagnet (trident) building blocks periodically arranged in a perpendicular fashion (Fig. 1a). Each nanomagnet is small enough to be single-domain and elongated, so that the magnetization points toward one of two possible directions along the long axis of each nanomagnet, thus representing a single Ising-type moment. As these moments couple via dipolar magnetic fields, we refer to this system as the dipolar trident lattice. Using synchrotron-based photoemission electron microscopy PEEM^22^ (Methods section), we directly visualize thermally induced magnetic relaxation of the trident lattice, and demonstrate the inability of the system to access a fully ordered state down to temperatures around 150 K, when tuning the balance of competing interactions. We show how, above 150 K, the ordering preferences of the system can be altered between two long-range ordered phases via an intermediate disordered state, exhibiting a continuous presence of vertex defects, which through their migration control the relaxation process and configurational fluctuations in thermal equilibrium. Upon cooling, the disordered phase also evolves toward long-range order, exhibiting a mixture of the two magnetic configurations.

Energetically, moment configurations in the trident lattice (Fig. 1b) can be characterized by four vertex types^1, 9^ listed with increasing dipolar energy in Fig. 1c. In addition to the vertex types, the so-called trident types need to be taken into account, which are listed with increasing energy as Type A, B, and C in Fig. 1c. In order to understand the concept of competing interactions in the trident lattice, one has to be aware of the consequence of dipolar interactions: First, at four-nanomagnet vertices (for example, *α*, *δ*, *ε*, and *η* in Fig. 1a), nearest neighbors will preferably exhibit a head-to-tail moment alignment, giving rise to Type I vertices (Fig. 1c) and vortex-like states (Fig. 1d). Second, the tridents (*α*, *β*, and *γ* in Fig. 1a) favor an anti-parallel moment alignment (Fig. 1d). In a long-range picture, a system where vertex interactions are mostly minimized (Type I vertex domination) cannot satisfy all trident interactions, as Type B tridents will dominate the configuration landscape (Fig. 1e). In contrast, if trident interactions are minimized (Type A trident domination), the energetically higher Type II vertices will exhibit a dominating presence (Fig. 1f). In other words, it is impossible to simultaneously satisfy both vertex interactions and trident interactions and the system is expected to be frustrated.

### Direct observation of thermal relaxation

**Fig. 2:**
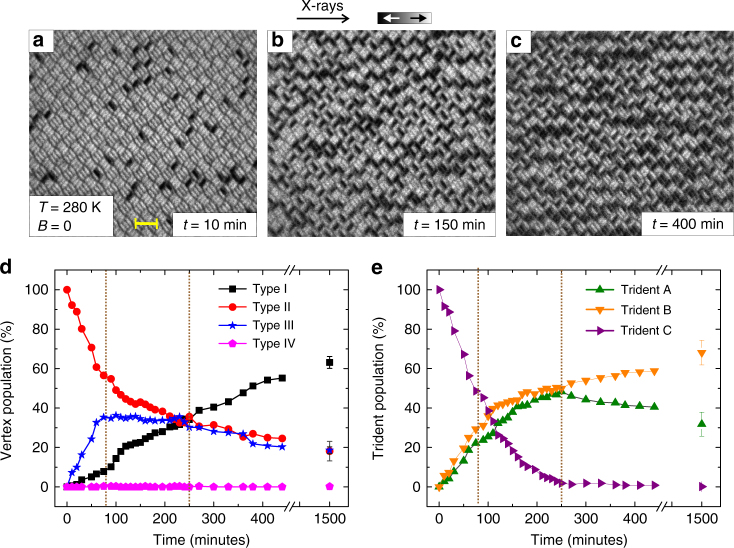
Thermal relaxation of the dipolar trident lattice. **a**–**c** XMCD images of the dipolar trident lattice undergoing thermally induced magnetic relaxation from a magnetically saturated state to a low-energy equilibrium configuration. The yellow scale bar indicates a length of 1 µm. **d** Experimentally obtained temporal evolution of vertex-type population extracted from XMCD images recorded at a constant temperature (*T* = 280 K). **e** Trident population plotted for the same relaxation process

As a first step, we aim to visualize the consequence of geometrical frustration on the ordering mechanism in a trident lattice consisting of nanomagnets with length, width, and thickness of 450, 150, and 2.7 nm, respectively (Methods section). The lattice spacing was chosen, so that the two relevant parameters *a* and *b* (Fig. 1a), which control the strength of trident and vertex interactions, respectively, are set to be 50 nm each. The blocking temperature *T*_B_, which we define as the temperature at which moment re-orientations start to occur within the timescale needed to acquire a single-PEEM image (7–9 s per image)^9, 23^ was determined to be 270 K. The sample was kept at a constant temperature of 280 K and a saturating magnetic field (*B* = 30 mT) was shortly applied along the incoming X-ray direction. After the field is switched off, the system undergoes thermally induced magnetic relaxation from a well-defined energetically excited state toward a highly disordered equilibrium state (Fig. 2a–c; Supplementary Fig. 1 and Supplementary Movie 1).

A quantitative analysis of the relaxation mechanism is obtained by looking at the vertex-type and trident-type populations plotted as a function of time (Fig. 2d, e). Starting from a 100% Type II vertex and Type C trident background (saturated state), the system experiences a rapid drop and rise in Type II and Type III vertex populations, respectively, while Type I vertices are moderately on the rise (Fig. 2d). In parallel, the population of Type C tridents decreases rapidly, while Type A and B tridents are showing an almost equal increase (Fig. 2e). As the system continues to relax, the high number of generated Type III vertex defects converts into Type I vertices, while new defects are continuously generated with an ongoing decrease in Type II vertices. Thus, the system stagnates in terms of Type III population during this stage (Fig. 2d). Finally, the system enters a stage where the Type I vertex population rises continuously at the cost of Type II and Type III vertices, until equilibrium is achieved. Kinetic Monte Carlo simulations (Methods section)^19^ are in good agreement with the experimental observations (Supplementary Fig. 2).

### Controlling the balance of competing interactions

**Fig. 3:**
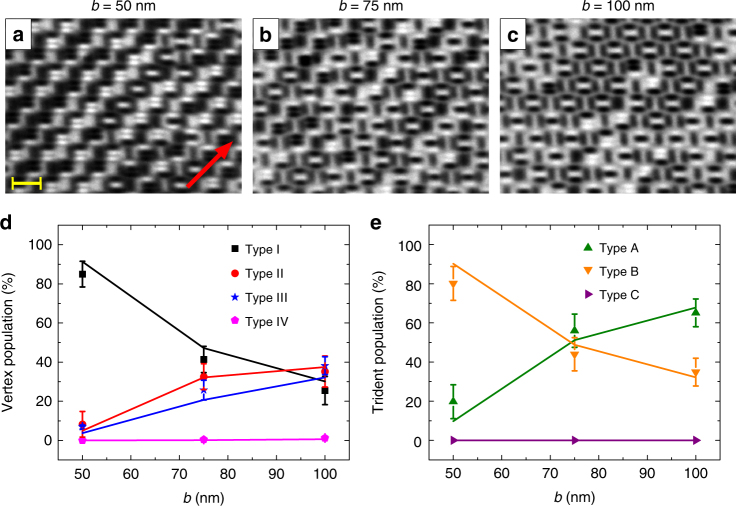
Tuning geometrical frustration in dipolar trident lattice. **a**–**c** XMCD images of equilibrium configurations of trident lattices with various lattice spacings (*a* = 50 nm = const, *b* = 50, 75, and 100 nm) recorded at *T* = 300 K (blocking temperature *T*_B_ = 310 K). The red arrow indicates the direction the X-ray propagation vector. The yellow scale bar indicates a length of 1 µm. **d** Experimentally observed vertex-type population plotted as a function of lattice parameter *b*. **e** Trident-type population plotted with increasing lattice parameter *b*. Experimental observations (filled symbols) are in satisfactory agreement with equilibrium Monte Carlo simulations (lines). The results reveal the transition from a Type I vertex and Type B trident-ordered state (*b* = 50 nm), to a disordered configuration (*b* = 75 nm) with no clear ordering preference, and, finally, to a Type A trident-ordered state (*b* = 100 nm), as the lattice parameter *b* is increased. The error bars represent standard deviations originating from ten experimental observations

The dominance of Type I vertices for *a* = *b* = 50 nm indicates that the competition between vertex interactions and trident interactions is not perfectly balanced and, as a result, a high degree of frustration is not obtained. This balance of competing interactions can be tuned by varying the *b*/*a* ratio. Therefore, a second set of trident arrays are fabricated (Methods section), where *a* = 50 nm is set to stay constant, while *b* is varied to be 50, 75, and 100 nm. The sample was kept at a constant temperature of 330 K (*T*_B_ = 310 K) for ~24 h before it was cooled down to 300 K and magnetic images were obtained (Fig. 3a–c; Supplementary Fig. 3). Plotting the vertex populations and trident populations as a function of *b* (Fig. 3d, e), we see a transition from a largely ordered phase with Type I vertex and Type B trident domination (*b* = 50 nm), through a disordered phase with short-range order and no clear preference for any vertex types (*b* = 75 nm), to, finally, a phase that shows trends toward Type II vertex and Type A trident preference (*b* = 100 nm).

This balancing act between competing trident interactions and vertex interactions indicates that accessible low-energy states can be directly tuned by a variation of the *b*/*a* ratio. This is a direct consequence of the enforced lattice constraints, making it impossible for the involved entities to simultaneously minimize both trident interactions and vertex interactions. Similar to previous work^24, 25^, calculating the energy spectrum of an isolated five-nanomagnet building block (Supplementary Fig. 4), clarifies the degeneracies listed in Fig. 1e, f. For a system with *b*/*a* = 1, the building block ground state is four-fold degenerate, with a clear gap (Δ*E* in Supplementary Fig. 4) to the second energy band, which consists of eight quasi-degenrate states. This gap can be tuned by varying the *b*/*a* ratio. The critical ratio *b*/*a* = 1.5 of the relevant lattice parameters can be further comprehended, when comparing the dipolar energies for fully ordered magnetic configurations of Type A/Type II and Type B/Type I trident types and vertex types as a function of *b*, while *a* is kept constant at 50 nm. This is shown in Supplementary Fig. 5, where dipolar energies are equalized, when *b* reaches a value around 75 nm. In other words, this is the point where the dipolar trident lattice reaches maximum degeneracy with no preference for any of the twelve states listed in Fig. 1e, f.

### Low-temperature configurations and magnetic structure factors

As a next step, we study how the degeneracy of low-energy building block states affects moment configurations at lower temperatures. Previous work on highly frustrated artificial kagome spin ice^17, 24, 26^ showed that despite an extensive degeneracy and short-range ordering at higher temperatures, the long-range nature of dipolar interactions gives rise to ordered configurations at lower temperatures. Therefore, it is our purpose here to see whether any signatures of long-range ordering can be observed in the dipolar trident lattice, particularly in the case of highest degeneracy, when *b*/*a* = 1.5.

**Fig. 4:**
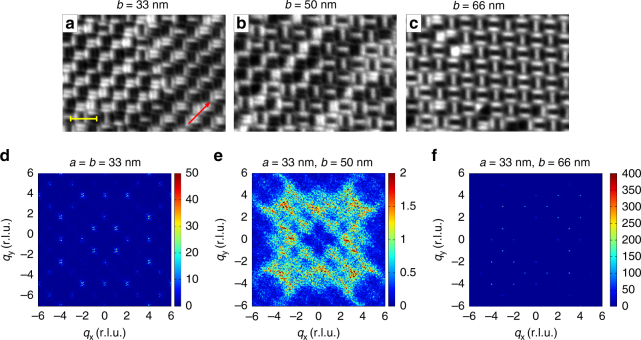
Experimental low-temperature observations. **a**–**c** XMCD images of equilibrium configurations of the dipolar trident lattice with various lattice spacings (*a* = 33 nm = const, *b* = 33, 50, and 66 nm) recorded at *T* = 150 K (blocking temperature *T*_B_ = 160 K). The red arrow indicates the direction the X-ray propagation vector. The yellow scale bar indicates a length of ~600 nm. **d**–**f** Magnetic scattering patterns of moment configurations achieved in dipolar trident lattices with lattice parameters **d** *a* = 33 nm and *b* = 33 nm, **e** *a* = 33 nm and *b* = 50 nm, and **f** *a* = 33 nm and *b* = 66 nm, following thermal annealing. While long-range order with relatively sharp peaks is seen for *b* = 33 nm and *b* = 66 nm, the diffuse patterns for *b* = 50 nm indicate highly disordered moment configurations with short-range correlations

We prepared another set of trident lattices, consisting of nanomagents with lengths *L* = 300 nm, widths *W* = 100 nm, and thickness *d* = 2.4 nm, together with the corresponding lattice parameters *a* = 33 nm = const. and *b* = 33, 50, and 66 nm. This reduction of nanomagnet size resulted in a lowering of the blocking temperature down to 160 K. The sample was kept in vacuum at room temperature for 20 days, before it was cooled down to 150 K for X-ray magnetic circular dichroism (XMCD) image acquisition (Fig. 4a–c). While long-range ordered patterns are observed for *b* = 33 and 66 nm, the *b* = 50 nm array remains disordered at 150 K. Similar to the room-temperature data (Fig. 3), tuning of geometrical frustration can again be inferred from the evolution of vertex populations and trident populations as a function of lattice parameter *b* (Supplementary Fig. 6). Our results thus indicate that for the case *b*/*a* = 1.5, the system is caught in a short-range ordered phase, while both the *b*/*a* = 1 and 2 cases exhibit long-range ordered ground state configurations.

A deeper quantitative insight into the experimentally accessed low-temperature configurations is achieved by calculating the respective magnetic structure factors^15^ (Methods section), which are shown in Fig. 4d–f. For both *b* = 33 nm and *b* = 66 nm (Fig. 4d, f), we see relatively sharp peaks in the magnetic structure factors. The splitting of the magnetic peaks into four satellites for *b* = 33 nm reflects multi-domain long-range ground state ordering for *b* = 33 nm (Fig. 4d). Figure 4f (b = 66 nm) shows sharp magnetic peaks that stand for an almost single-domain long-range ordered ground state consisting mostly of a tile of two Type A tridents, as can also be seen from real-space images (Fig. 4c). However, a dramatic change in the magnetic structure factor is observed for the lattice parameter combination of *a* = 33 nm and *b* = 50 nm (Fig. 4e), where the diffuse patterns indicate the presence of a disordered phase consisting of a complex arrangements of possible low-energy configurations (Fig. 1e, f), where neither of these states dominate. Similar patterns are also observed in the structure factor of the simulated *b* = 50 nm system (Supplementary Fig. 7). To ensure that these simulated configurations are in fact in thermal equilibrium, we use the parallel tempering technique^27–29^ (Methods section), where the equilibration time is estimated by calculating the so-called exponential autocorrelation time^29^, *τ*_exp_, which itself is defined by the temporal decay of the autocorrelation function, Γ ∝ exp[−*t*/*τ*_exp_] (Methods section). The agreement between experimental and simulated configurations provides evidence that the experimental observations also represent states in thermal equilibrium. For comparison, we also calculated the magnetic structure factor of a purely paramagnetic trident lattice (Supplementary Fig. 8). In this case, no peaks appear in the structure factor map, providing further evidence that our dipolar trident lattice with *a* = 33 nm and *b* = 50 nm is in a disordered phase, where none of the predicted ground states is able to dominate the landscape at 150 K. While only partial order is able to set in at 150 K with relatively weak peak intensities, magnetic structure factor simulations clearly show that the peak intensities rise with decreasing temperature (Supplementary Fig. 9). Eventually, the system evolves toward long-range order at low temperatures, exhibiting a mixture of ordered stripes (Supplementary Fig. 10), which consist of a tile of Type A and Type B tridents and a mixture of Type I and Type II vertices. Compared to classical artificial square ice, which exhibits trivial ground state ordering, the mixed phase patterns in the dipolar trident lattice reflect the high degree of frustration in this geometrically frustrated magnetic metamaterial. For interested readers, a comparison of experimental observations to artificial square ice can be found in Supplementary Note 1.

## Discussion

In summary, we presented a magnetically frustrated metamaterial, which provides the possibility to directly control competing dipolar interactions at the nanoscale, thus allowing versatile tuning of geometrical frustration and ground state configurations. The complex phase into which the system gets trapped, when competing interactions are balanced, opens up multiple questions regarding the physics of the dipolar trident lattice, in particular the question regarding possible phase transitions toward complex long-range ordered states at lower temperature regimes^17–19^. Experimentally, this will require the fabrication of trident lattices consisting of nanomagnets with lateral dimensions that go beyond the spatial resolution of known magnetic imaging techniques^17^, and will therefore rely on emerging scattering and spectroscopic techniques^17, 26, 30, 31^.

## Methods

### Sample fabrication

Similar to previous work^5, 23^, dipolar trident lattices were fabricated by taking advantage of lift-off-assisted electron-beam lithography: a silicon (100) substrate was first spin-coated with a 70-nm-thick layer of polymethylmethacrylate resist. Then, trident lattices with various lattice spacings were defined onto the sample with a VISTEC VB300 electron beam writer. Next, using a Semicore SC600 e-beam evaporator, a ferromagnetic permalloy (Ni_80_Fe_20_) film was deposited at a base pressure of 1.2 × 10^−7^ Torr, which was followed by lift-off in acetone at a temperature of 50 °C. Thermally driven moment fluctuations in one set of artificial spin ice samples were realized by fabrication of ultrathin nanomagnets with length *L* = 450 nm and width *W* = 150 nm. The samples discussed in this work had thicknesses of 2.7 nm and 3 nm, resulting in blocking temperatures of 270 and 310 K, respectively. For low-temperature measurements, the blocking temperature was moved down to 160 K by preparing nanomagnets with lengths, widths, and thickness of 300, 100, and 2.4 nm, respectively.

### Photoemission electron microscopy

Measurements were performed using the cryogenic photoemission electron microscope PEEM3 at beamline 11.0.1 at the Advanced Light Source^22^. Magnetic images were captured by taking advantage of XMCD at the Fe L_3_-edge^32^. The obtained contrast is a measure of the projection of the magnetization on the X-ray polarization vector, so that nanomagnets with a magnetization parallel or antiparallel to the X-ray polarization either appear black or white. Nanomagnets with moments having ±45° and ±135° angles with respect to the incoming X-rays appear dark and bright, respectively.

### Magnetic structure factor

The magnetic structure factor is calculated as

1 $$I\left( {\bf{q}} \right) = \frac{1}{N}\mathop {\sum }\limits_{i = 1}^N \mathop {\sum }\limits_{j = 1}^N {\bf{S}}_i^ \bot \cdot {\bf{S}}_j^ \bot {\rm{exp}}\left( {i{\bf{q}} \cdot {\bf{r}}_{i,j}} \right),$$

where ${\bf{S}}_i^ \bot = {\bf{S}}_i\! -\! \left( {\widehat {\bf{q}} \cdot {\bf{S}}_i} \right)\widehat {\bf{q}}$ is the component of the spin vector of each island perpendicular to the reciprocal space vector **q**, the unit vector is given by $\widehat {\bf{q}} = {\bf{q}}{\mathrm{/}}\left\| {\bf{q}} \right\|$, **r**_*i*,*j*_ is the vector from island *i* to *j*, and *N* is the total number of islands. Equation (1) has the same form as in neutron scattering experiments and has previously been used to analyze artificial spin-ice configurations^15^.

### Simulations

We model each nanomagnet as an infinitesimally thin compass needle with a uniform magnetic moment density $\frac{{\left| m \right|}}{L}$. The magnetic moment points along the long axis of the island. This description is equivalent to placing a magnetic charge at each end of the island^5, 19, 33^. The inter-island interaction is given by the Hamiltonian

2 $$H_{{\mathrm{ij}}} = \frac{{\mu _0\left| m \right|^2}}{{4\pi L^2}}\left[ {\frac{1}{{\left| {r_{a_{\mathrm{i}}}\! -\! r_{a_{\mathrm{j}}}} \right|}} - \frac{1}{{\left| {r_{a_{\mathrm{i}}}\! -\! r_{b_{\mathrm{j}}}} \right|}} - \frac{1}{{\left| {r_{b_{\mathrm{i}}}\! -\! r_{a_{\mathrm{j}}}} \right|}} + \frac{1}{{\left| {r_{b_{\mathrm{i}}}\! -\! r_{b_{\mathrm{j}}}} \right|}}} \right],$$

where $r_{a_{\mathrm{i}}}$ and *r*_bi_ are the locations of the positive and negative magnetic charge on the *i*th nanomagnet, *μ*_0_ is the magnetic permeability, *L* is the island length, and |*m*| = *MV* is the magnetic moment of each nanomagnet with *M* being the saturation magnetization and *V* the nanomagnet volume. The system size is 1200 islands, and only interactions with a magnitude of at least 2% of the nearest-neighbor interaction are included in the simulation (~35 neighbors per spin).

To simulate the dynamics of the system, we use the kinetic Monte Carlo method^9, 23^, which evolves the system through single-spin flips. A particular spin flip move is selected with a probability proportional to its rate. Assuming an Arrhenius-type switching behavior, the rate of a spin flip is given by *v* = *v*_0_ exp(−*E/k*_B_ *T*), where *k*_B_=8.62 × 10^−5^ eV K^−1^ is the Boltzmann constant, *ν*_0_ is the so-called attempt frequency, *T* is the temperature, and *E* is the reorientation barrier, which is equal to the intrinsic energy barrier *E*_0_ plus half the dipolar energy gain associated with moment re-orientations (Eq. (2)). The simulation parameters *M* = 240 kA m^−1^, *E* = 0.887 eV, and *ν*_0_ = 10^12^ s^−1^ were fit using the experimental relaxation results of Fig. 2. These values are in good agreement with previous studies on thermally activated artificial spin ice^5, 9, 23^. In addition to the assumption of a uniform system, where all nanomagnets have the same intrinsic energy barrier, we also investigated the role of disorder^9^. This is included by assuming a random variation in *E*_0_, which follows a Gaussian distribution with mean *E*_0_ = 0.893 eV and standard deviation *σ* = 0.05 eV (Supplementary Fig. 2).

To generate equilibrium configurations, for the results presented in Fig. 3 and structure factor calculations shown in Supplementary Fig. 7, we use the parallel tempering technique^27, 28^. Replicas of the system are simulated at a number of temperatures simultaneously using kinetic Monte Carlo. After every Monte Carlo sweep a move is proposed which swaps the configuration of a pair of replicas at neighboring temperatures *T*_*n*_ and *T*_*m*_. This move is accepted with a probability

3 $$P_{{\rm{swap}}}\left( {n,m} \right) = {\rm{min}}\left\{ {1,{\rm{exp}}\left[ { - \left( {\frac{1}{{k_{\rm{B}}T_m}} - \frac{1}{{k_{\rm{B}}T_n}}} \right)\left( {E_n - E_m} \right)} \right]} \right\},$$

where *E*_*n*_ is the energy of replica *n*. The set of temperatures is selected such that the acceptance ratio of a swap move at each temperature is greater than 0.2. A value of *M* = 362 kA m^−1^ is used to obtain the results in Fig. 3. The equilibration time is estimated with the exponential autocorrelation time^29^, *τ*_exp_. This is defined by the decay of the autocorrelation function, Γ ∝ exp[−*t*/*τ*_exp_]. It is calculated for the autocorrelation function of the spin overlap function between two concurrent independent simulations, its absolute value, and the configuration energy throughout the parallel tempering simulation. Taking the largest of these calculated exponential autocorrelation times, the first 20 × *τ*_exp_ time steps are treated as equilibration time and discarded.

### Code availability

Codes for numerical calculations in this study are available from the corresponding author upon reasonable request.

### Data availability

Data supporting the findings in this study are available from the authors upon request.

## Electronic supplementary material

Supplementary Information

Description of Additional Supplementary Files

Supplementary Movie 1
