# Author Correction: Nanoscale control of competing interactions and geometrical frustration in a dipolar trident lattice

**DOI:** 10.1038/s41467-017-02139-2

**Published:** 2017-12-12

**Authors:** Alan Farhan, Charlotte F. Petersen, Scott Dhuey, Luca Anghinolfi, Qi Hang Qin, Michael Saccone, Sven Velten, Clemens Wuth, Sebastian Gliga, Paula Mellado, Mikko J. Alava, Andreas Scholl, Sebastiaan van Dijken

**Affiliations:** 1 Advanced Light Source, Lawrence Berkeley National Laboratory (LBNL), 1 Cyclotron Road, Berkeley, CA 94720 USA; 2 COMP Centre of Excellence, Department of Applied Physics, Aalto University, P.O. Box 11100, Espoo FI-00076 Aalto, Finland; 3 Molecular Foundry, Lawrence Berkeley National Laboratory (LBNL), 1 Cyclotron Road, Berkeley, CA 94720 USA; 4 Dipartimento di Fisica, Università di Genova, via Dodecaneso 33, I-16146 Genova, Italy; 5 NanoSpin, Department of Applied Physics, Aalto University School of Science, P.O. Box 15100, FI-00076 Aalto, Finland; 6 Department of Physics, University of California, Santa Cruz, CA 95064 USA; 7 Materials Sciences Division, Lawrence Berkeley National Laboratory, 1 Cyclotron Road, Berkeley, CA 94720 USA; 8 Institut für Nanostruktur- und Festkörperphysik, Universität Hamburg, Jungiusstrasse 11, 20355 Hamburg, Germany; 9 Center for X-ray Optics, Lawrence Berkeley National Laboratory, 1 Cyclotron Road, Berkeley, CA 94720 USA; 10 Daegu Gyeongbuk Institute of Science and Technology (DGIST), 50-1 Sang-ri, Hyeonpung-myeon, Dalseong-gun, Daegu, 42988 Republic of Korea; 11 SUPA, School of Physics and Astronomy, University of Glasgow, Glasgow, G12 8QQ UK; 12 School of Engineering and Sciences, Adolfo Ibáñez University, Diagonal Las Torres, 2640 Peñalolén, Santiago Chile

*Nature Communications* **8**:995 10.1038/s41467-017-01238-4; Article published online: 17 October 2017

The original version of this article contained an error in the legend to Figure 4. The yellow scale bar should have been defined as ‘~600 nm’, not ‘~600 µm’. This has now been corrected in both the PDF and HTML versions of the article.

