# Supplementary material for: Nanoscale control of competing interactions and geometrical frustration in a dipolar trident lattice
Source: Nat Commun. 2017 Oct 17;8:995. doi: 10.1038/s41467-017-01238-4 (PMC5727135; doi:10.1038/s41467-017-01238-4)
Supplement: Supplementary file 1 — Supplementary Information [file 41467_2017_1238_MOESM1_ESM.pdf]

Supplementary Information

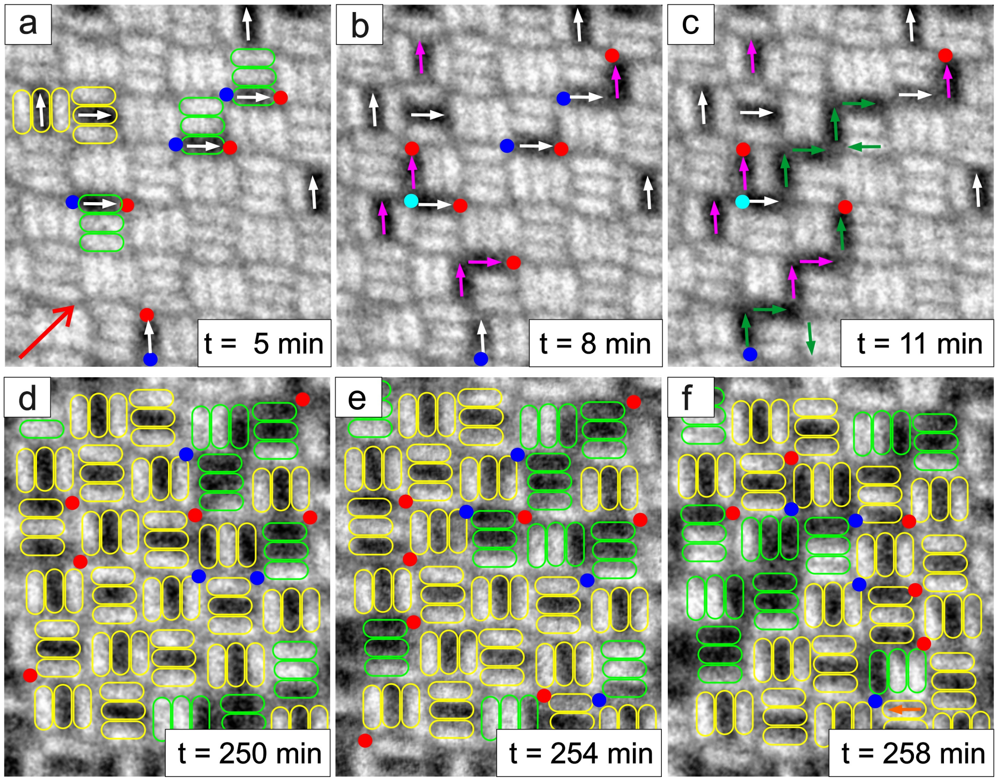

Supplementary Figure 1. Vertex defect migration for a system with lattice parameters *a* = *b* = 50 nm. a-c, During the initial relaxation stage, isolated Type III defects (blue and red circles representing $-2q$ and $+2q$ charges, respectively) are created which can then either separate via sequential moment reversal or get interconnected via annihilation of neighboring defect pairs. Eventually Type IV vertices (cyan blue circle representing a $-4q$ charge) are created, which branch in two directions towards Type III defects (two $+2q$ charges). **d-f,** At later stages, Type A trident domains (yellow frames), that dominate during the stagnation regime, are taken over by Type B tridents (green frames). The continuing shift of domains is triggered by repeated creation, motion and annihilation of Type III vertex defects (blue and red dots) along the domain boundaries.

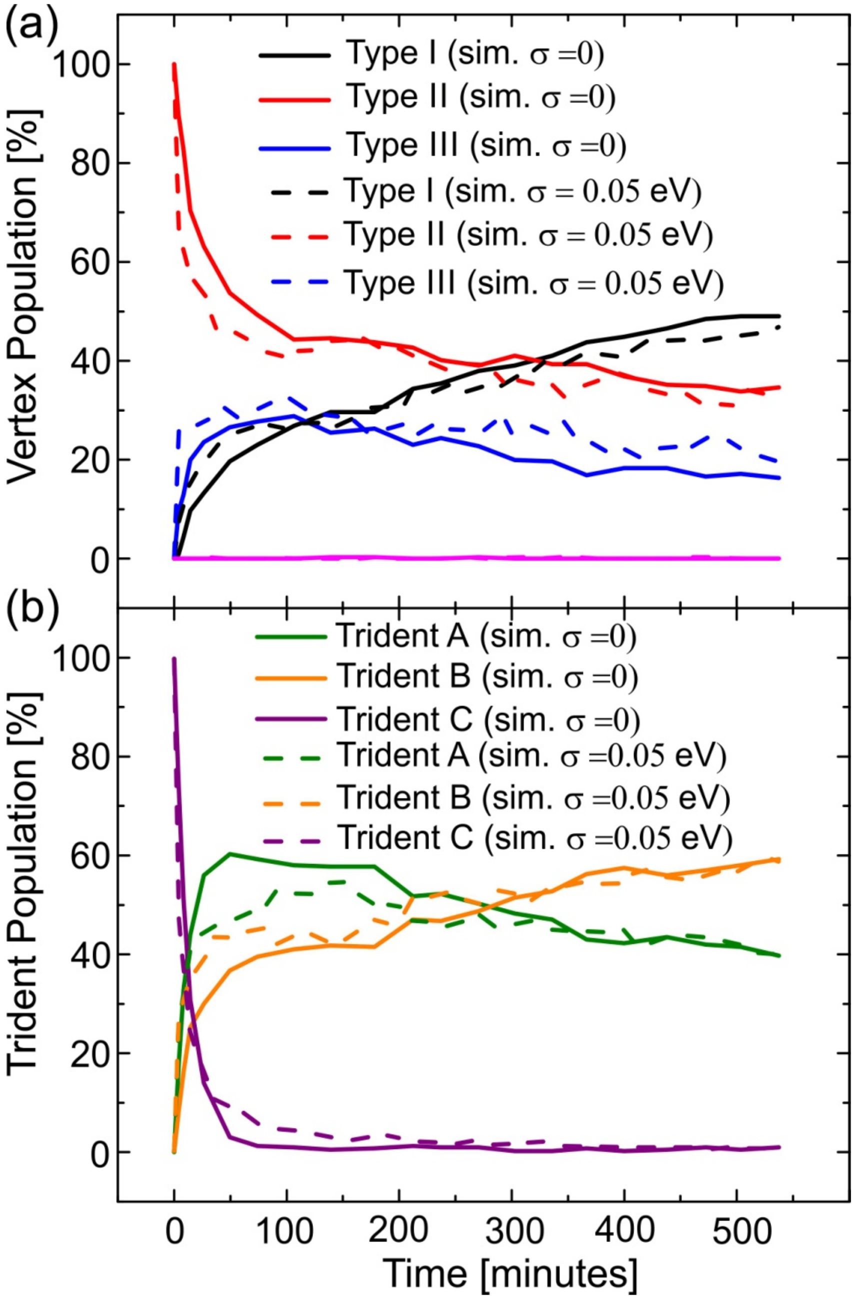

Supplementary Figure 2. Simulated magnetic relaxation of the dipolar trident lattice with *a* = *b* = 50 nm. a-b, Kinetic Monte Carlo simulation data of vertex- and trident type populations, respectively, plotted as a function of time.

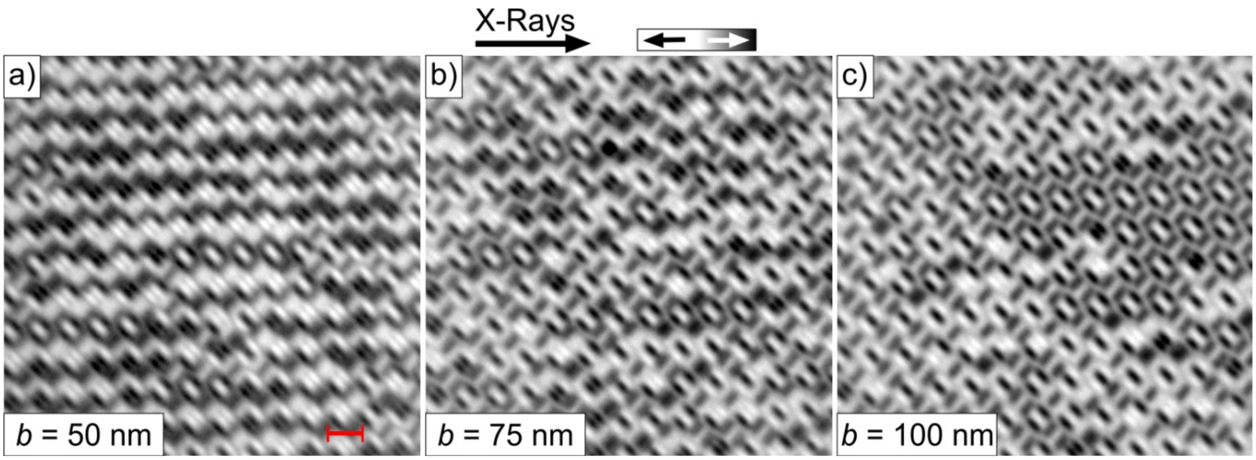

Supplementary Figure 3. Tuning geometrical frustration in the dipolar trident lattice. a-c, Zoomed out XMCD images (field of view = 12 µm) of the dipolar trident system with varied lattice parameters (*a* = 50 = *const*. and *b* = 50, 75 and 100 nm, respectively). The red scale bar indicates a length of 1 µm.

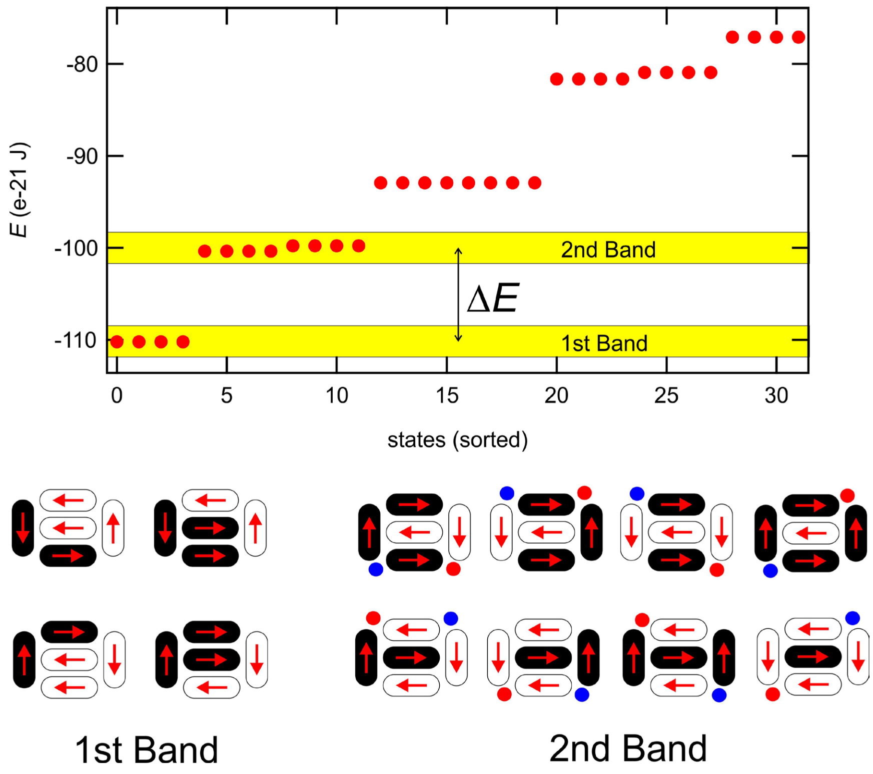

Supplementary Figure 4. Energy spectrum of 5-nanomagnet building block. Using the dipolar needle approximation (see methods), dipolar energies corresponding to all possible 2^5 = 32 states of a five-nanomagnet building block in the trident lattice (assuming $a = b$ = 50 nm) are calculated. The energy gap ΔE between the 4-fold degenerate ground state configurations (1st Band) and the quasi 8-fold degenerate states in the 2nd Band can be directly varied by changing the lattice parameter ratio b/a.

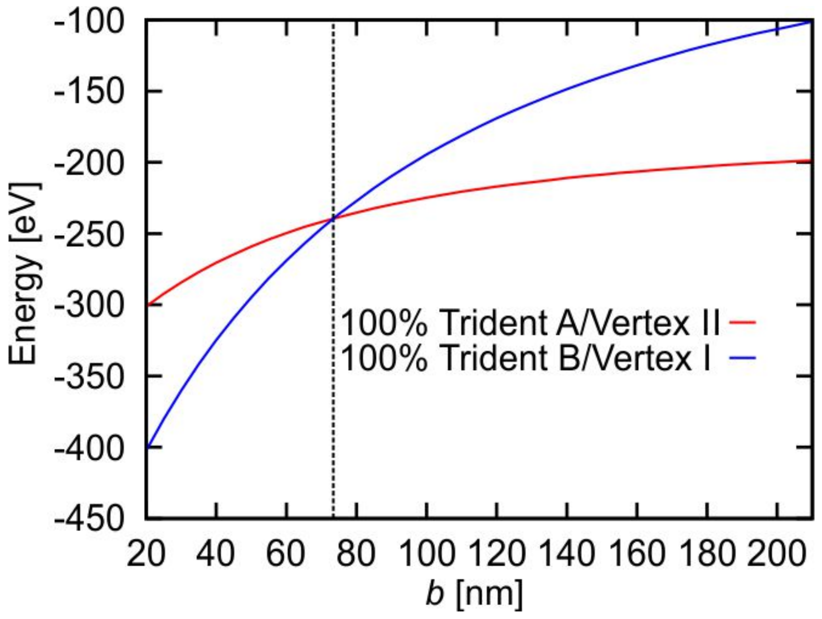

Supplementary Figure 5. Balancing competing interactions. Energy of ordered configurations in different lattice geometries, plotted as a function of a varying lattice parameter b, while a = 50 nm is kept constant. The energies are equal for $b = 75$ nm ($^{b}/_{a}$ = 1.5), indicating that competing trident and vertex interactions are equalized for this lattice parameter.

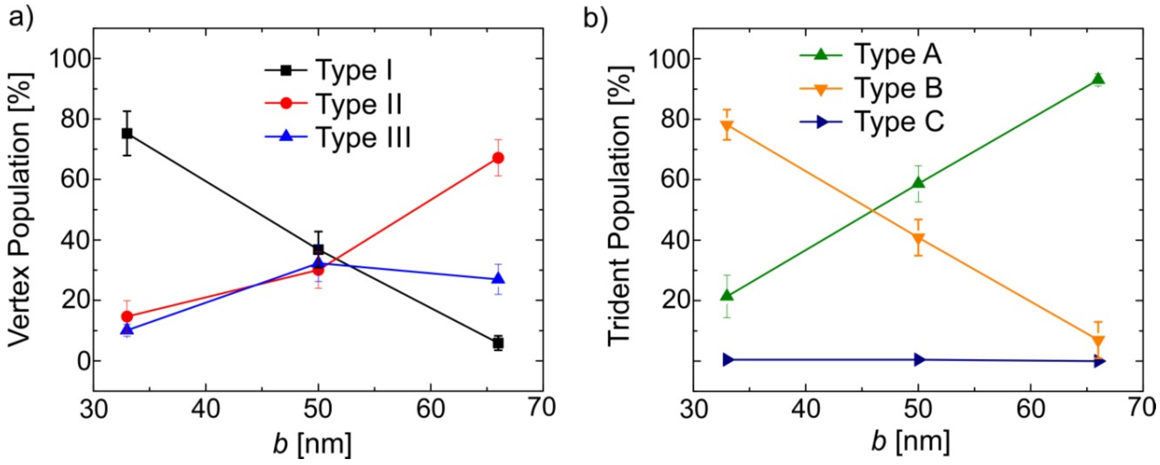

Supplementary Figure 6. Low energy magnetic configurations at 150 K. a and **b,** Average vertex- and trident populations recorded at T = 150 K and plotted as a function of lattice parameter b. The error bars stand for standard deviations originating from ten observations.

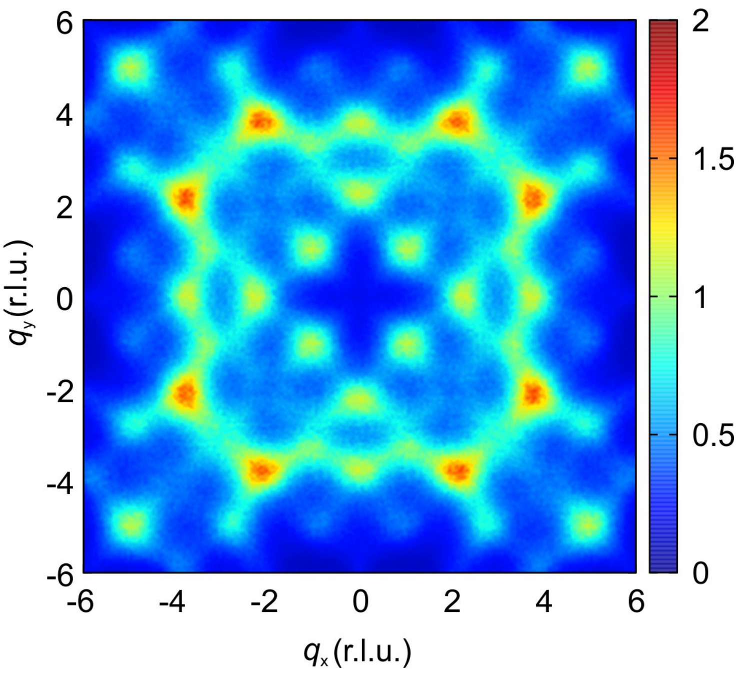

Supplementary Figure 7. Simulated diffuse magnetic structure factor at 150 K. Magnetic structure factor generated for the simulated equilibrium configurations at 150 K (see methods) of the dipolar trident lattice with lattice parameters a = 33 nm and b = 50 nm. A value of M = 200 kA m^{-1} is used. The plotted data are an average from 100 configurations.

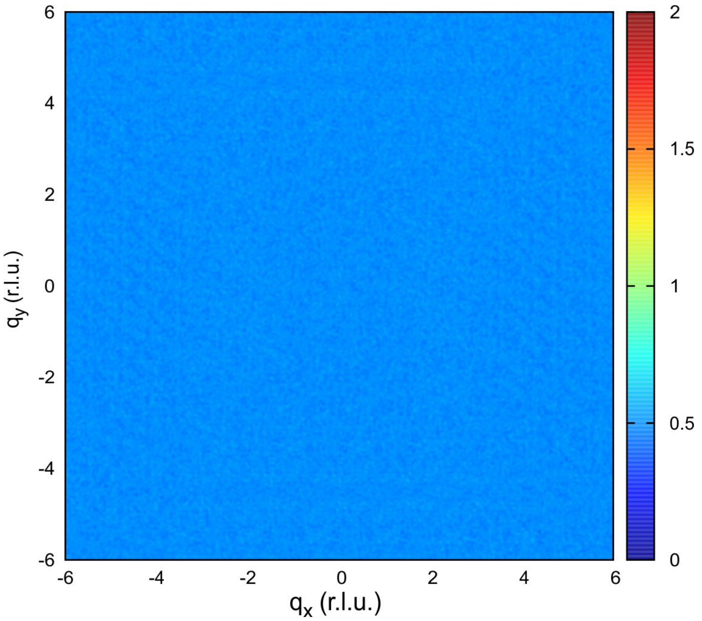

Supplementary Figure 8. Simulated magnetic structure factor of a paramagnetic trident lattice. Magnetic structure factor generated for fully randomized paramagnetic moment configurations.

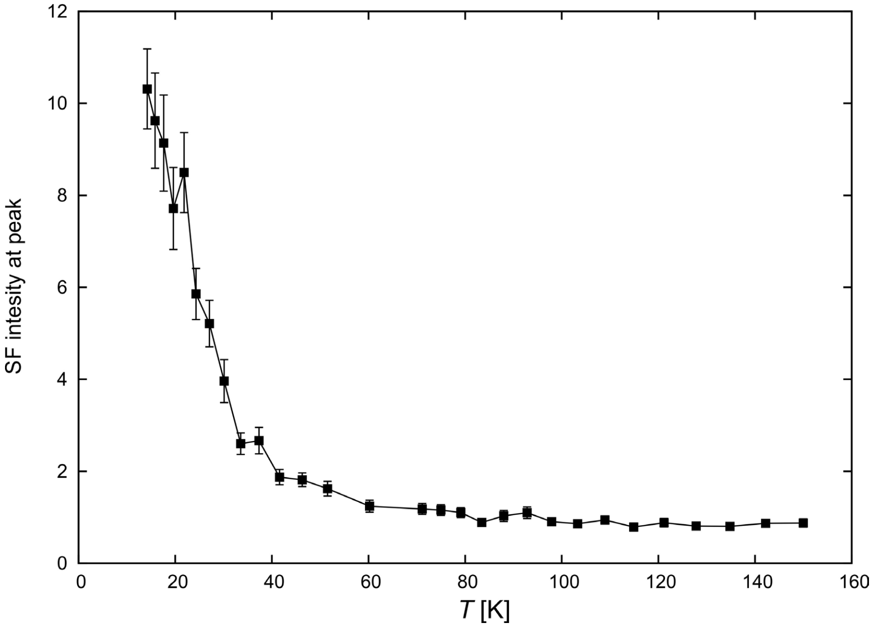

Supplementary Figure 9. Simulated peak intensity as a function of temperature. Simulated magnetic structure factor intensity at $q_x = q_y = 1.5$ as a function of temperature. The error bars stand for standard deviations originating from 100 simulated configurations at each given temperature.

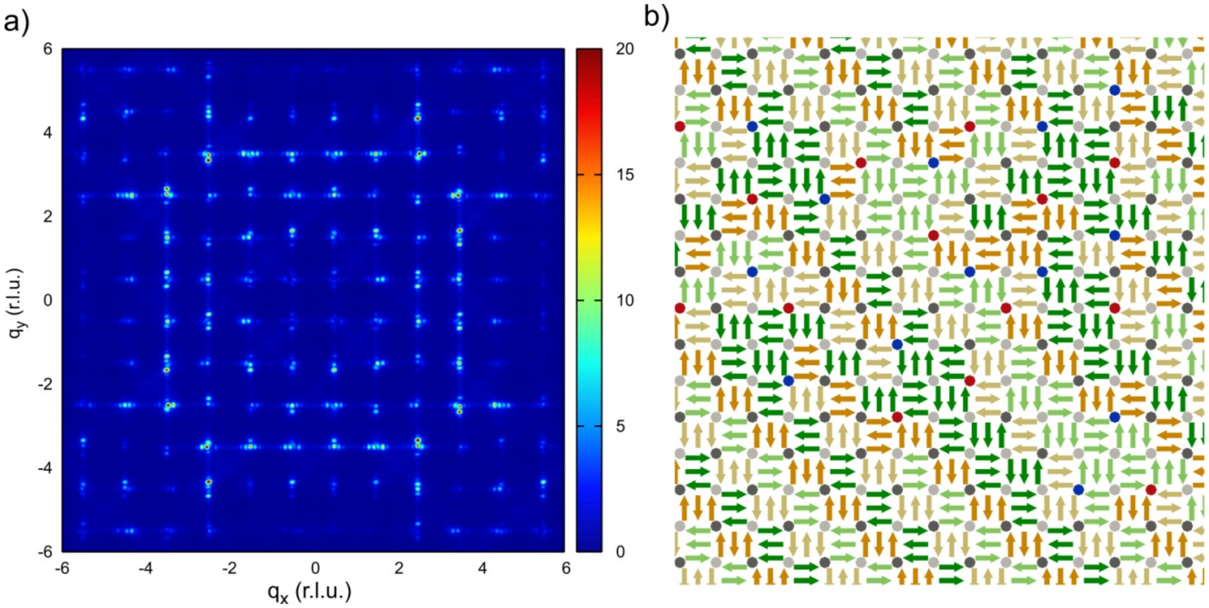

Supplementary Figure 10. Simulated configurations at 15 K. a, Magnetic structure factor generated for the simulated equilibrium configurations at 15 K (see methods) of the dipolar trident lattice with lattice parameters a = 33 nm and b = 50 nm. A value of M = 200 kA m^{-1} is used. The plotted data are an average of 100 configurations. **b,** Snapshot of simulated moment configurations achieved at 15 K showing ordered stripe patterns. The vertices are colored by type (light grey = type I, dark grey = type II, red or blue = type III) and the spins are colored by trident type (orange = type A, green = type B).

Supplementary Note 1: Comparison to Artificial Square Ice

An interesting comparison to quasi-three-dimensional artificial square ice[1] emerges, when removing contributions of the central β-nanomagnets (see Fig. 1a) and mapping the remaining moments onto a perfect square lattice. In this case, comparable scattering patterns as observed for square ice configurations with certain height offsets[1] (below the critical offset) between neighboring nanomagnets are obtained (see Supplementary Fig. 11). However, the absence of pinch-point singularities, compared to the case of three-dimensional artificial square ice with a critical height offset[1], indicates that the dipolar trident lattice does not exhibit a macroscopic degeneracy, but will access a long-range ordered state at lower temperatures. This assertion is supported by the simulated magnetic configurations that are obtained at 15 K (see Supplementary Figs. 9 and 10).

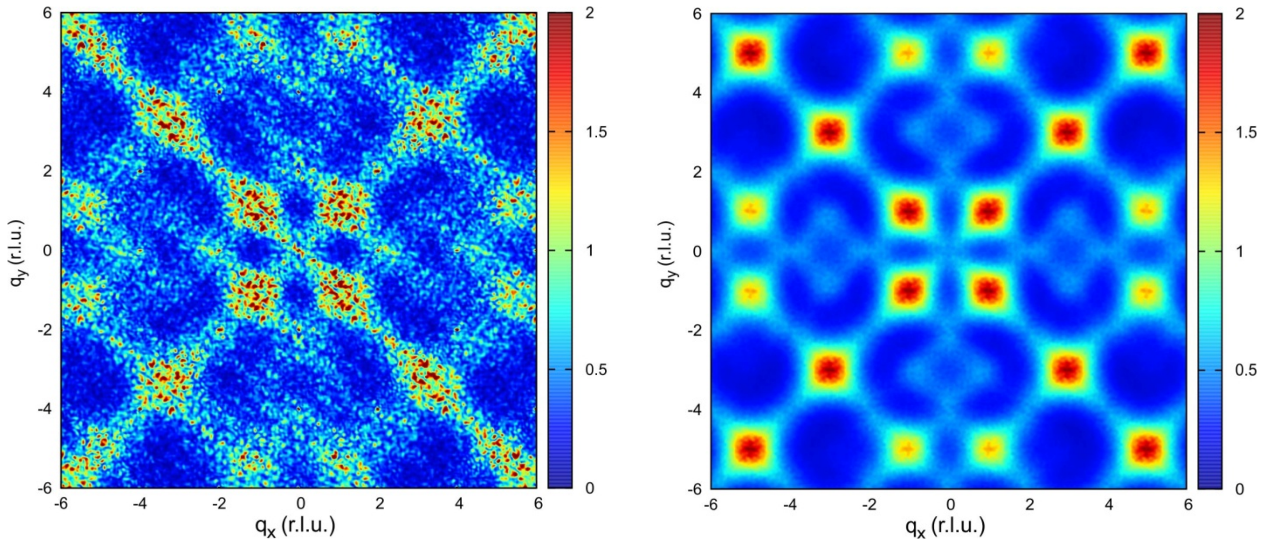

Supplementary Figure 11. Magnetic structure factor of moment configurations mapped onto a square lattice. (left panel) Experimentally measured magnetic structure factor of a trident lattice ($a = 33$ nm and $b = 50$ nm) at 150 K, where contributions of β-nanomagnets are removed and the remaining moments are placed onto a perfect square lattice. (right panel) Simulated configurations at the same temperature (see also main Fig. 4 for comparisons).

Supplementary References

1. Perrin Y, Canals B, Rougemaille N. Extensive degeneracy, Coulomb phase and magnetic monopoles in artificial square ice. *Nature* **540**, 410-413 (2016).
