# Supplementary material for: Nanoscale control of competing interactions and geometrical frustration in a dipolar trident lattice
Source: Nat Commun. 2017 Oct 17;8:995. doi: 10.1038/s41467-017-01238-4 (PMC5727135; doi:10.1038/s41467-017-01238-4)
Supplement: Supplementary file 2 — Description of Additional Supplementary Files [file 41467_2017_1238_MOESM2_ESM.pdf]

Description of Additional Supplementary Files

File Name: Supplementary Movie 1
Description: Thermally induced magnetic relaxation of the dipolar trident lattice. XMCD image sequence (9 minutes per image) obtained at T = 280 K of dipolar trident lattice undergoing thermal relaxation from an energetically excited state towards a multidomain low-energy state.
